# Supplementary material for: Dedicated transcriptomics combined with power analysis lead to functional understanding of genes with weak phenotypic changes in knockout lines
Source: PLoS Comput Biol. 2020 Nov 12;16(11):e1008354. doi: 10.1371/journal.pcbi.1008354 (PMC7685438; doi:10.1371/journal.pcbi.1008354)
Supplement: S1 Fig — (PDF) [file pcbi.1008354.s001.pdf]

Supplemental Figure S1

Alignments of the ORF regions of *A930004D18Rik* and *A830005F24Rik* among the mouse species and closely related outgroup species where the sequences could be identified in the respective genomic regions. (A) shows the summary of the alignments. (B), (C), and (D) show the alignments of *A930004D18Rik* ORF1, *A930004D18Rik* ORF2, and *A830005F24Rik* ORF separately. For each alignment, all nucleotide sequences have been aligned to the one of *Mus musculus* as reference, and amino acids have been translated according to the reference frame. Alignment was done in Geneious Prime (2019.0.3 Biomatters Ltd.). The topology of the species tree is shown at the left part. Nucleotides and amino acids are shown in IUPAC codes. Enablers (common disablers) are marked with red rectangles.

A

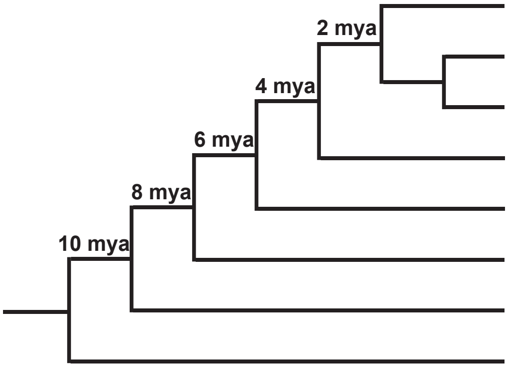

	A930004D18Rik ORF1		*A930004D18Rik* ORF2		*A830005F24Rik* ORF	
	Intact ORF	Youngest enabler	Intact ORF	Youngest enabler	Intact ORF	Youngest enabler
Mus musculus	YES	YES	YES	YES	YES	YES
Mus spretus	YES	YES	NO	YES	NO	YES
Mus spicilegus	YES	YES	NO	YES	NO	YES
Mus caroli	NO	NO	NO	YES	NO	YES
Mus mattheyi	NO	NO	NO	YES	NO	YES
Mus pahari	NO	NO	NO	YES	NO	NO
Apodemus uralensis	NO	NO	NO	NO	NO	NO
Rattus norvegicus	NO	NO	NO	NO	NO	NO
**Inferred emergence time**	2 - 4 mya		8 - 10 mya		6 - 8 mya	

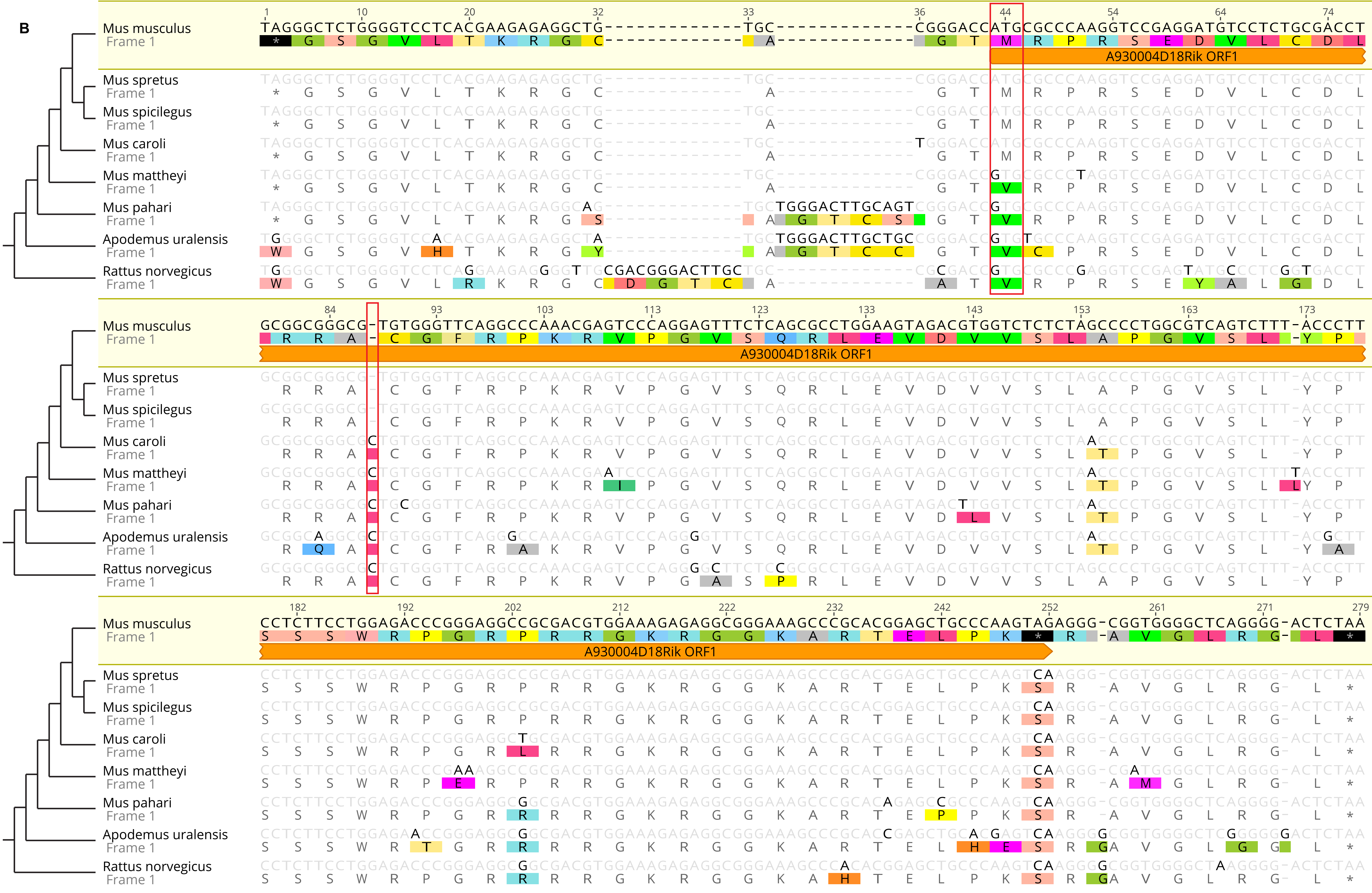

B
Mus musculus
Frame 1
A930004D18Rik ORF1
Mus spretus
Mus spicilegus
Mus caroli
Mus mattheyi
Mus pahari
Apodemus uralensis
Rattus norvegicus

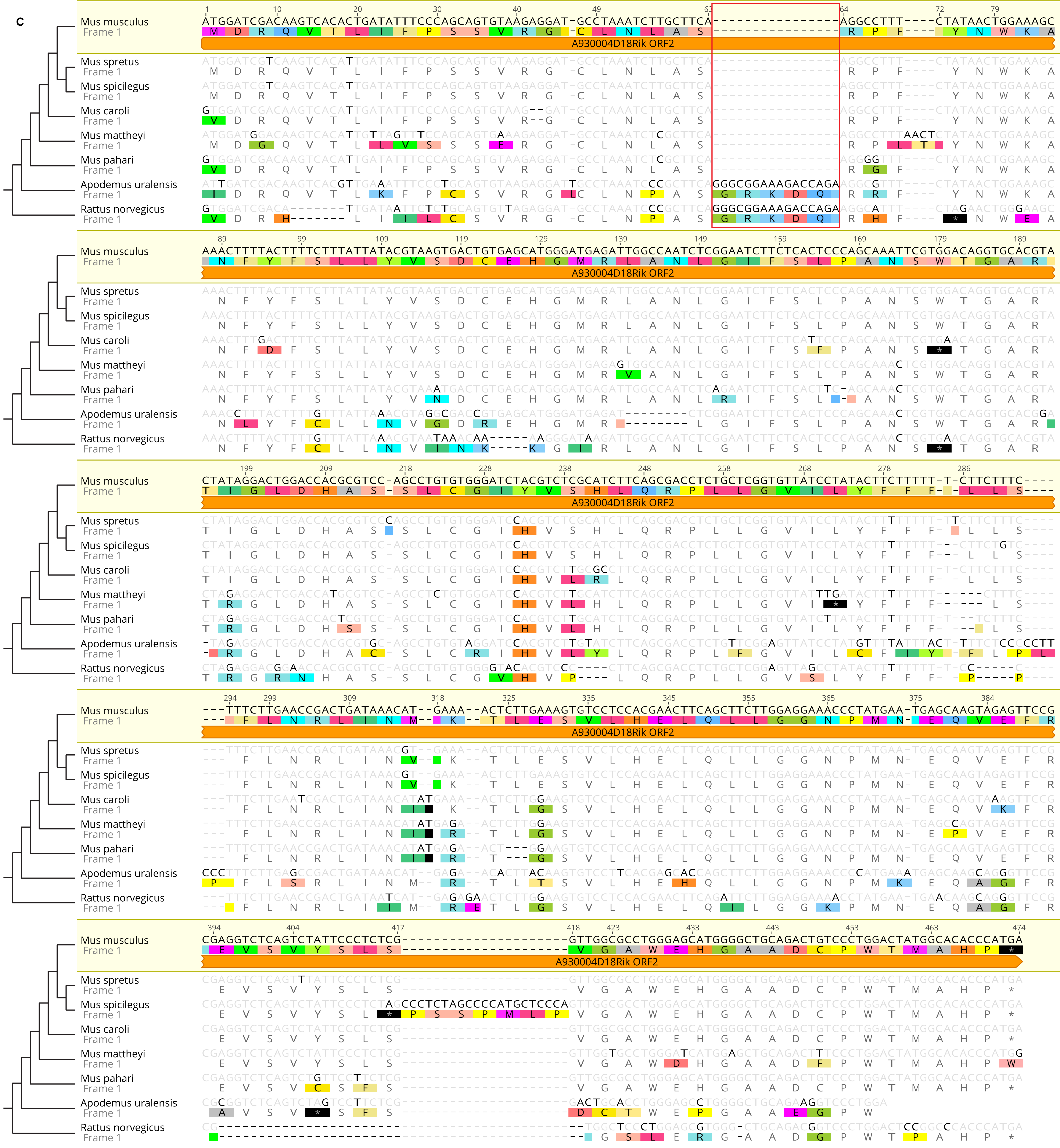
C
Mus musculus
Mus spretus
Mus spicilegus
Mus caroli
Mus mattheyi
Mus pahari
Apodemus uralensis
Rattus norvegicus
Frame 1
A930004D18Rik ORF2

D

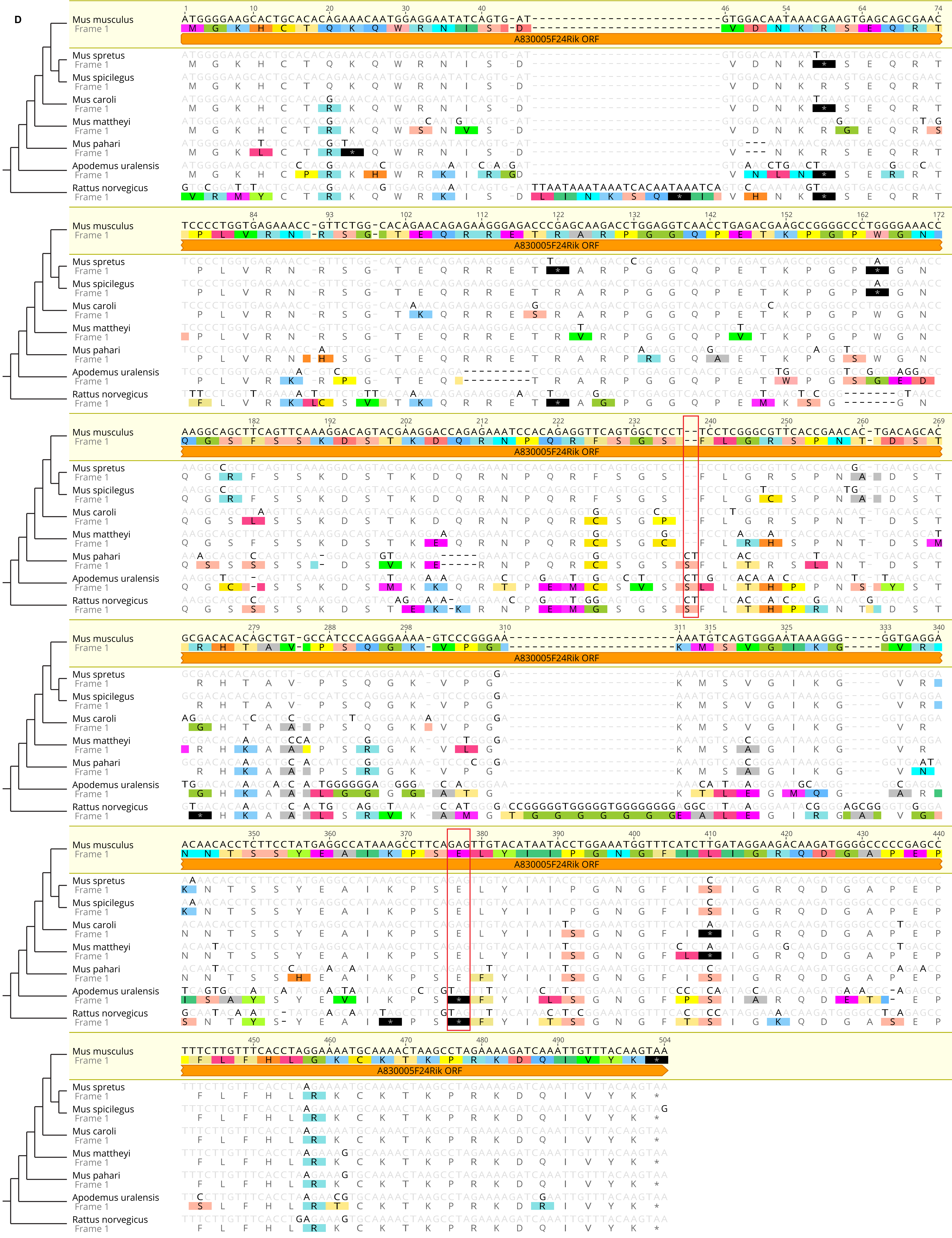

Mus musculus
Frame 1
A830005F24Rik ORF
Mus spretus
Mus spicilegus
Mus caroli
Mus mattheyi
Mus pahari
Apodemus uralensis
Rattus norvegicus
