# Supplementary material for: Dedicated transcriptomics combined with power analysis lead to functional understanding of genes with weak phenotypic changes in knockout lines
Source: PLoS Comput Biol. 2020 Nov 12;16(11):e1008354. doi: 10.1371/journal.pcbi.1008354 (PMC7685438; doi:10.1371/journal.pcbi.1008354)

Supplemental Figure S2.

Results of PCA analysis with limb lengths. The three panels from top to bottom show the six PCs with explained variances.

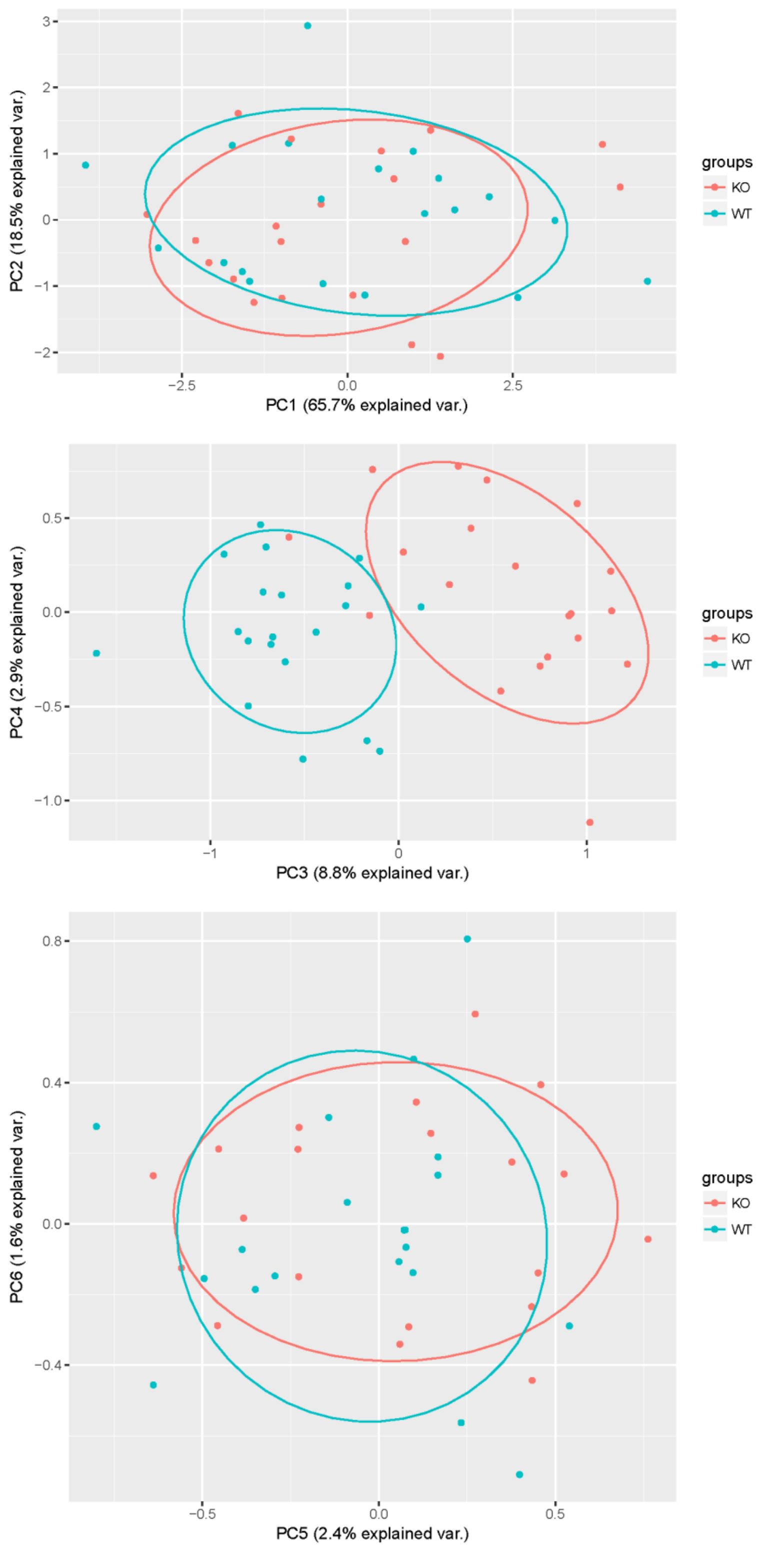

Supplement: S2 Fig — (PDF) [file pcbi.1008354.s002.pdf]
