# Supplementary material for: Dedicated transcriptomics combined with power analysis lead to functional understanding of genes with weak phenotypic changes in knockout lines
Source: PLoS Comput Biol. 2020 Nov 12;16(11):e1008354. doi: 10.1371/journal.pcbi.1008354 (PMC7685438; doi:10.1371/journal.pcbi.1008354)
Supplement: S1 File — (DOCX) [file pcbi.1008354.s015.docx]

**Supplemental Methods**

*Analysis of the data from International Mouse Phenotyping Consortium and preliminary tests for A830005F24Rik knockout line*

We downloaded all available phenotypic data of *A830005F24Rik* knockout line through the International Mouse Phenotyping Consortium (IMPC, http://www.mousephenotype.org/) web service (15 Jun. 2016). In total, 267 parameters were measured for this line, 44 of them were filtered out due to quality issue, intermediate values, redundancy, and descriptive information. For each of the rest 223 parameters, if it is categorical, we fitted the data to a logistic linear model: “Measurement ~ Genotype + Gender”, and calculated its P-Value with R function “glm”; if it is numerical, then if it is without an increment value (time series; one mouse was measured several times at different time points), we fitted the data to a linear model: “Measurement ~ Genotype + Gender”, and calculated its P-Value with R function “lm”; if it is with an increment value, we fitted the data to a linear mixed-effects model: “Measurement ~ Genotype + Gender + (1|Subject) + (1|Increment)” with package “lme4” (1.1.14) [1], and then we calculated its P-Value by performing a likelihood ratio test of this model against a null model:

Measurement ~ Gender + (1|Subject) + (1|Increment) with R function “anova”. After calculating all P-Values, we performed Benjamini-Hochberg FDR correction with R function “p.adjust”. In the end, we found 11 parameters with P-Values less than or equal to 0.05 (S3 Table). Note that the wildtypes measured by IMPC are wildtype C57BL/6N mice in IMPC, but not from the same knockout litters.

In order to support the preliminary results for the *A830005F24Rik* line from IMPC, we performed eight preliminary tests on small numbers of animals (S3 Table). For all the mice used for preliminary tests, they were genotyped in advance, age matched between knockouts and wildtypes. The genotypes were then masked to the experimenters.

For body length, heart weight normalized against body weight, and red blood cell count, we always did the experiments in the morning and did the measurements immediately after euthanizing the mice. We measured the body length and heart weight normalized against body weight according to the IMPC protocols. We counted the red blood cells with a hemocytometer after preparing blood collected from retro-orbital puncture and then diluted in PBS with EDTA solution.

For ultrasonic vocalization test (USV), we followed the method described in [2]. In brief, young adult female and male mice were held in individual cages with a 12-h light/dark cycle, and females were primed with regular male bedding two weeks before measurements. Sound recordings were conducted inside a USV recording box with four separate compartments. Each compartment was fitted with one ultrasound-microphone and all four microphones were connected to a multichannel recording device. Two pairs of mice were measured at the same time (“Group” in S3 Table), and the female and the male in the same pair had same genotype. The order of the pairs to be measured was randomly shuffled. All the recordings started at 19:00 for each measurement, and the number of songs for each mouse in the first three hours was analyzed.

The elevated plus maze test, open field test, and novel object test are described in Methods (*Behavioral tests*). For light/dark box and startle test, the general experimental set-ups and conditions are same as the other three tests. The light/dark box and startle test was performed in the same arena as the open field test. In addition to the arena, a black plastic tool box was used as a shelter measuring 25 cm wide x 40 cm long. The box had two openings at the center where the mouse could exit the box. The arena was placed directly under the camera. At the beginning of the experiment, the mouse was placed inside the box using a transparent plastic transfer pipe. The light/dark box test lasted 5 minutes. During this time, the first time the mouse looked out of the shelter (nose visible) and the first time the mouse left the shelter completely (complete mouse visible) were measured. If the mouse never looked out of the shelter or never left the shelter, this was scored as 300 seconds by the experimenter. During the second part of the test, a key chain was dropped from a height of 136 cm next to the test arena. During the next 5 minute period, the first time the mouse looked out of the shelter (nose visible) and the first time the mouse left the shelter completely (complete mouse visible) were measured. If the mouse never looked out of the shelter or never left the shelter, this was scored as 300 seconds by the experimenter. At the end of the experiment, the arena and the shelter were cleaned with 30% ethanol. For all the mice used in the four behavioral tests, they stayed in individual cages with a 12-h light/dark cycle, and all females were kept in a room with only females and all males were kept in a room with only males at least two weeks before measurements. All the tests were performed between 8:00 and 12:00. The mice with the same gender were experimented for the same test in the same morning, and the order of the mice to be measured was randomly shuffled.

The results of the preliminary tests are summarized in S3 Table. For body length and red blood cell count, our preliminary observations were even in the opposite directions of the results from IMPC. Therefore, we did not follow up these phenotypes. For heart weight normalized against body weight, we found the method to measure the heart weight was inaccurate because the amount of blood left in each heart varied largely among different samples. For the ultrasonic vocalization test, we found the song numbers among different groups varied a lot for unknown reasons. In addition, the song numbers within each group also varied strongly, partially due to the unsuccessful synchronization of the estrous cycles of females, which not only affected the signals from females but also affected the signals from males because the female and male belonging to one pair could interact with each other during the whole experiment. For the four behavioral tests, we found all the data from female mice had large variation, probably due to that they were in the different stages of estrous cycle. Considering that our main purpose was to show the phenotypic changes in knockout mice, and it would take lots of effort to control the stages of female estrous cycle, we decided to only focus on male data and only perform further behavioral tests on males. For the elevated plus maze test, we discovered the time tendency described in the Results, and continued the test with expanded samples. For the open field test and novel object test, we did not discover any obvious differences of the parameters. For the light/dark box and startle test, we found the measurements were either showing large variation (nose time, nose time after startle) partially due to the inaccurate observation of tiny dark nose in black background (box), or not informative at all (mouse time , mouse time after startle).

**References**

1. Bates D, Mächler M, Bolker B, Walker S. Fitting Linear Mixed-Effects Models Usinglme4. Journal of Statistical Software. 2015;67(1). doi: 10.18637/jss.v067.i01.

2. von Merten S, Hoier S, Pfeifle C, Tautz D. A role for ultrasonic vocalisation in social communication and divergence of natural populations of the house mouse (Mus musculus domesticus). PLoS One. 2014;9(5):e97244. doi: 10.1371/journal.pone.0097244. PubMed PMID: 24816836; PubMed Central PMCID: PMCPMC4016290.
