# Supplementary material for: Dedicated transcriptomics combined with power analysis lead to functional understanding of genes with weak phenotypic changes in knockout lines
Source: PLoS Comput Biol. 2020 Nov 12;16(11):e1008354. doi: 10.1371/journal.pcbi.1008354 (PMC7685438; doi:10.1371/journal.pcbi.1008354)
Supplement: Attachment — Submitted filename: Response_to_reviewers.docx [file pcbi.1008354.s019.docx]

William Stafford Noble, PhD

Deputy Editor

PLOS Computational Biology

Dear Dr. Noble,

Thank you for handling our manuscript and organizing constructive reviews. Following the referee’s suggestions, we further revised the manuscript. New results are presented in the text, Fig 2D and 2E, Table 4, and the relating supporting information. We list detailed point-to-point responses to the review comments below.

Reviewer #1: This study assesses the potential for transcriptomic analysis of mouse knockout strains with no physiological phenotype. The study is straightforwardly designed and the paper is well organized. The authors provide a systematic analysis of differential expression analysis, assessing the power to detect differentially expressed genes for multiple sample sizes. Importantly, multiple methods are used and results are clear and consistent. The authors also describe their choice in knockout genes, using an unbiased approach to potentially discover the functions of two uncharacterized genes.

The main outcomes of the paper are a demonstration that knockout strains may exhibit transcriptomic differences, even in cases without “overt” phenotypes, and a power analysis of expected effects. The first outcome strongly depends on what a researcher considers an “overt” phenotype, which are often extreme outcomes obtained from screening (e.g. lethality). That such knockouts nevertheless exhibit transcriptional effects is not surprising, but the power analysis presented in this context is informative. However, the authors do not discuss any of the existing literature on power analysis in transcriptomic studies, even while using a standard software tool to address this problem in their analysis, so it is unclear how special the mouse knockout case is in terms of power analysis.

> We appreciate the reviewer’s insightful comments. Statistics for RNA-Seq studies, especially case-control designs, has been well established [1, 2], and its power analysis has also been well discussed and studied theoretically [3-6]. However, serious practical studies about the power analysis with real data are still lacking, and it is one of the major contributions of our manuscript in the field. Parameters in the experimental designs of RNA-Seq studies, especially sample size, have been ignored in most studies. For example, in the large-scale transcriptomic studies conducted by DMDD which we cited in the Introduction [7], the sample sizes of the 73 knockout lines range from 2.5 (2 vs. 3) to 12.0 (8 vs. 16), which leads to quite large difference in the power to detect differentially expressed genes. If a study only looks for the genes with dramatically differential expression, *i.e.*, genes with fold change larger than 1.5 or 2, the parameter choice of experimental design might not be crucial; but for the genes with small differential expression, such as the cases focused in our manuscript, a carefully choosing parameter set would be important. We further clarified this in the text.

Specific comments:

1. The title is not informative and borderline nonsensical. The introductory quote is only marginally helpful, and the metaphor remains opaque. A more scientifically precise title would be helpful.

> We have now better explained why we have chosen this metaphor. In fact, Wolpert´s quote was very important at its time and it was often repeated at conferences. It was rather instrumental to release the money for building up today´s extensive phenotyping pipelines. Hence, we think it is appropriate to keep it in the title. However, we have extended the title in a more technical manner.

2. The manuscript could benefit from stronger conclusions and recommendations, especially regarding power analysis of knockout mouse strains. I believe this is the most important topic in the paper.

> Thanks very much for the suggestion. We further emphasized this in the Conclusions.

3. The abstract refers to “one gene” and “the other”, which is a bit confusing. Although the genes do not have the most conversant names, it would be helpful for reader comprehension and future researchers if the genes were named in the Abstract.

> We changed them to gene symbols.

4. I question that it is appropriate to call transcriptomic studies of knockouts a “new approach” (Page 3) after almost 20 years of such studies.

> We agree that transcriptomic studies of knockouts were done since almost 20 years, but RNA-Seq started to be applied on knockouts only in the past ten years. It is more sensitive than microarray-based techniques. However, serious power analysis to detect weak difference has not been applied on practical data, which means the analysis part of the RNA-Seq-based approach is still new. Anyway, we changed the wording in the text.

5. The paper consistently refers to “weak” phenotypes without explicitly defining what is meant by “weak”. Gene expression itself is a phenotype, which is presumably “strong” when thousands of genes are changing expression. I understand that this language reflects how many researchers tend to discuss their knockout studies, but this paper should precisely define what is meant.

> Weak phenotypes mean the phenotypic difference detected by traditional phenotyping tests is small. We clarified this in the text.

6. How generalizable are these results beyond gene knockouts? While knockouts are a classic experiment to determine gene function, much current modeling work is focused on the effects of variants (both coding and noncoding) that do not entirely ablate a gene’s transcript. This is particularly important when assessing the function of disease variants from human GWAS of complex diseases, where an overt disease phenotype is not expected but transcriptomic changes may inform on modifications of disease-relevant processes.

> Thanks very much for the suggestion. We believe our transcriptome analyses and results, especially the power analysis, also suit for all transcriptome case-control studies. For more complicated transcriptome experiments, similar extended power analysis could also be performed, but this is beyond the scope of our manuscript. We added relevant discussion in the text.

7. P6: "We guess that IMPC conducted this knockout strategy..." Were the IMPC contacted in any attempt to validate this guess?

> We did contact both IMPC and EMMA (the European Mouse Mutant Archive, from which the mutant line was obtained), but were only provided with the information of the general targeting strategies of IMPC which can also be publicly found, including the information at “https://www.mousephenotype.org/about-impc/targeting-strategies/” and [8-12]. There was no information we could get about this specific line, *i.e.*, our guess could not be validated. We clarified this in the text.

8. P 12: Bonferroni correction is possibly too conservative for transcriptome data, as genes are not independently expressed in tissues.

> We agree that Bonferroni correction is too conservative for transcriptome data, and it is only used for the theoretical analysis in Fig 2A, and we used FDR correction (BH) for all other analyses. However, FDR correction is based on the P-Value distribution of all the tested genes, which is not suitable for this particular analysis, and then we use Bonferroni correction instead. Even the multiple testing method we used for this theoretical analysis is too strict, we can still get very important information from the result: genes with very low fold changes can be detected when the condition of other parameters (such as sample size, read count, and dispersion) satisfies, even under strict multiple testing control. We clarified this in the text.

9. The origin of control mice was not clear. I assume wild-type littermates were used for comparisons, but this needs to be stated. Details on control mice, sample collection, and processing are essential to understand the power analysis and determine any potential batch effects.

> We did use the corresponding littermates for each comparison. We apologize that we did not state this clear in the original manuscript. We clarified this in the text.

Reviewer #2: The authors in this paper aim to characterise phenotypes of genes that have non-lethal phenotypes. To this end, they perform power analysis to identify the conditions, in which small levels of transcript changes can be traced. This is then applied to identify the function of two genes that do not yet have defined phenotypic effect upon knockout in mice. The authors carry out a multitude of behavioural phenotyping and observe modest effects. They further carry out transcriptional analysis to identify the changes in transcriptional networks upon knockout of the two candidate genes.

In the absence of overt phenotypes, the focus of the paper shifts to characterise the function of the two genes using transcriptomics approach. However, the authors do very little to elucidate the biological differences caused by gene knockouts using the transcriptomic data and leave much to be desired. The majority of the paper focuses on identification of optimal parameters in RNAseq analysis. The authors argue that for genes that have non-overt phenotypes more samples and deeper sequencing analysis is required to identify the underlying phenotype. To identify the optimal parameters the authors focus mainly on the number of DEGs. This is not an important criteria to be focusing on. The important criteria would be the underlying biological pathways the DEGs represent. At the least, the authors should perform enrichment analysis for each subsampling rather than reporting the number of DEGs. If the ultimate aim is to characterise the phenotype, additional analysis such as transcriptional activity analysis (using already available packages such as DoRothEA (Garcia-Alonso et al, 2019, Genome Research) can be performed. Network- propagation based approaches (again numerous approaches are available for transcriptomic datasets for this purpose) can further be used to identify the cellular pathways that are altered in knockout vs the control.

> Many thanks for the constructive comments and thus we carefully considered and conducted the suggested analyses.

We would like to point out that the number of differentially expressed genes (DEGs), which is the indicator of the power to detect DEGs, is the most important criteria to be focused on, because the DEGs and their statistics including P-Values, fold changes, and read counts, are the basis of downstream analysis. In other words, only if the DEGs are detected both specifically and sensitively enough, the downstream functional analysis can be valid. We further clarified this in the text. However, we agree on the comment that enrichment analysis should also be performed on the subsampling datasets. Hence, we performed GO enrichment analyses with GOseq [13] on the subsampling datasets of both sample size and sequencing depth (see Fig 2D and 2E, and the corresponding text). The proportion of overlapping GO terms between the significantly enriched terms (BH corrected P-Value ≤ 0.05) in the subsampling dataset and those in the full dataset was used as the measurement of the robustness, which is defined as the smaller ratio between the number of overlapping terms divided by the number of terms enriched in the subsampling dataset and the number of overlapping terms divided by the number of terms enriched in the full dataset. As described in the text, in general, the more sample or the deeper sequencing, the larger of the proportion of overlapping GO terms was discovered. In addition, the proportions of overlapping GO terms of the sample size subsampling datasets are smaller than those of the sequencing depth subsampling datasets, which indicates that raising the number of samples could be more efficient than raising sequencing depth for accurately detecting functional terms. Overall, the results of enrichment analyses on subsampling datasets are consistent with our previous subsampling results focusing on the numbers of DEGs, which is as expected.

The reason we used GOseq for our downstream functional analysis, which has been stated in our original text, is that GOseq is correcting for the selection bias in the RNA-Seq data. Specifically, the different lengths and expression levels of genes, combined as the different read counts of genes, cause different powers for each gene to be detected as DEGs. This selection bias has a strong effect on the accuracy of result, which has been carefully described and solved in [13]. However, few downstream functional analysis methods (including network-propagation based approaches) have taken this into serious consideration so far, just as few RNA-Seq studies have taken power analysis into serious consideration, which we have made a strong point in our original manuscript to remind people working in the field. The package for transcriptional activity analysis mentioned in the comment, DoRothEA, uses P-Values and log2 transformed fold changes as the input, which does not consider the selection bias of RNA-Seq either [14]. However, we still performed the analysis with DoRothEA on our datasets, and the results are presented in Table 4 and the relating text. We believe that this analysis does provide new useful information.

In addition, the authors explore the differentially expressed genes with fold change of 1.5, while mentioning that this space is rarely explored- this is not necessarily true. Standard practice in RNA-seq analysis is to use a combination of p-value and fold change cut off. While it is common for cut off of adjusted p-values to be 0.05, cut-offs on fold change values in RNA-seq analysis very much depend on the type of downstream analysis. Often studies will use less stringent log-fold changes and perform enrichment analysis to identify biological pathways.

> We believe this is a misunderstanding. We did not mean that the space with fold change of 1.5 was rarely explored. Instead, we meant that the space with fold change much smaller than 1.5 (down to 1.05) was rarely explored (Fig 2A). This refers to two aspects: (1) many studies only kept genes with fold changes larger than 1.5 (or 2) as DEGs for downstream analysis; (2) many studies did not set a fold change cutoff, but they anyway had no power to detect DEGs with low fold change due to small sample size or low sequencing depth in their RNA-Seq design. The DEGs with small fold changes can also be accurately detected and provide important information, which is more obvious for the case *A830005F24Rik* in our manuscript that all the DEGs in its datasets have small fold changes (Fig 3A).

I am recommending the paper be rejected at this point while suggesting to the authors to re-perform the analysis by focusing on if there biological pathways that are robustly identified regardless of sample size (e.g n=3 vs n=10) or sequencing depth.

> We have performed the suggested analysis and presented the results in the text. Please see our response above.

1.	Robinson MD, McCarthy DJ, Smyth GK. edgeR: a Bioconductor package for differential expression analysis of digital gene expression data. Bioinformatics. 2010;26(1):139-40. doi: 10.1093/bioinformatics/btp616. PubMed PMID: 19910308; PubMed Central PMCID: PMCPMC2796818.

2.	Anders S, Huber W. Differential expression analysis for sequence count data. Genome Biol. 2010;11(10):R106. doi: 10.1186/gb-2010-11-10-r106. PubMed PMID: 20979621; PubMed Central PMCID: PMCPMC3218662.

3.	Zhao S, Li CI, Guo Y, Sheng Q, Shyr Y. RnaSeqSampleSize: real data based sample size estimation for RNA sequencing. BMC Bioinformatics. 2018;19(1):191. doi: 10.1186/s12859-018-2191-5. PubMed PMID: 29843589; PubMed Central PMCID: PMCPMC5975570.

4.	Guo Y, Zhao S, Li CI, Sheng Q, Shyr Y. RNAseqPS: A Web Tool for Estimating Sample Size and Power for RNAseq Experiment. Cancer Inform. 2014;13(Suppl 6):1-5. doi: 10.4137/CIN.S17688. PubMed PMID: 25374457; PubMed Central PMCID: PMCPMC4213196.

5.	Li CI, Su PF, Guo Y, Shyr Y. Sample size calculation for differential expression analysis of RNA-seq data under Poisson distribution. Int J Comput Biol Drug Des. 2013;6(4):358-75. doi: 10.1504/IJCBDD.2013.056830. PubMed PMID: 24088268; PubMed Central PMCID: PMCPMC3874726.

6.	Li CI, Su PF, Shyr Y. Sample size calculation based on exact test for assessing differential expression analysis in RNA-seq data. BMC Bioinformatics. 2013;14:357. doi: 10.1186/1471-2105-14-357. PubMed PMID: 24314022; PubMed Central PMCID: PMCPMC3924199.

7.	Collins JE, White RJ, Staudt N, Sealy IM, Packham I, Wali N, et al. Common and distinct transcriptional signatures of mammalian embryonic lethality. Nature communications. 2019;10(1):2792. doi: 10.1038/s41467-019-10642-x. PubMed PMID: 31243271; PubMed Central PMCID: PMCPMC6594971.

8.	Pettitt SJ, Liang Q, Rairdan XY, Moran JL, Prosser HM, Beier DR, et al. Agouti C57BL/6N embryonic stem cells for mouse genetic resources. Nat Methods. 2009;6(7):493-5. doi: 10.1038/nmeth.1342. PubMed PMID: 19525957; PubMed Central PMCID: PMCPMC3555078.

9.	Bradley A, Anastassiadis K, Ayadi A, Battey JF, Bell C, Birling MC, et al. The mammalian gene function resource: the International Knockout Mouse Consortium. Mamm Genome. 2012;23(9-10):580-6. Epub 2012/09/13. doi: 10.1007/s00335-012-9422-2. PubMed PMID: 22968824; PubMed Central PMCID: PMCPMC3463800.

10.	Ryder E, Gleeson D, Sethi D, Vyas S, Miklejewska E, Dalvi P, et al. Molecular characterization of mutant mouse strains generated from the EUCOMM/KOMP-CSD ES cell resource. Mamm Genome. 2013;24(7-8):286-94. Epub 2013/08/06. doi: 10.1007/s00335-013-9467-x. PubMed PMID: 23912999; PubMed Central PMCID: PMCPMC3745610.

11.	Skarnes WC, Rosen B, West AP, Koutsourakis M, Bushell W, Iyer V, et al. A conditional knockout resource for the genome-wide study of mouse gene function. Nature. 2011;474(7351):337-42. Epub 2011/06/17. doi: 10.1038/nature10163. PubMed PMID: 21677750; PubMed Central PMCID: PMCPMC3572410.

12.	White JK, Gerdin AK, Karp NA, Ryder E, Buljan M, Bussell JN, et al. Genome-wide generation and systematic phenotyping of knockout mice reveals new roles for many genes. Cell. 2013;154(2):452-64. Epub 2013/07/23. doi: 10.1016/j.cell.2013.06.022. PubMed PMID: 23870131; PubMed Central PMCID: PMCPMC3717207.

13.	Young MD, Wakefield MJ, Smyth GK, Oshlack A. Gene ontology analysis for RNA-seq: accounting for selection bias. Genome Biol. 2010;11(2):R14. doi: 10.1186/gb-2010-11-2-r14. PubMed PMID: 20132535; PubMed Central PMCID: PMCPMC2872874.

14.	Garcia-Alonso L, Holland CH, Ibrahim MM, Turei D, Saez-Rodriguez J. Benchmark and integration of resources for the estimation of human transcription factor activities. Genome Res. 2019;29(8):1363-75. Epub 2019/07/26. doi: 10.1101/gr.240663.118. PubMed PMID: 31340985; PubMed Central PMCID: PMCPMC6673718.
