# Supplementary material for: Dedicated transcriptomics combined with power analysis lead to functional understanding of genes with weak phenotypic changes in knockout lines
Source: PLoS Comput Biol. 2020 Nov 12;16(11):e1008354. doi: 10.1371/journal.pcbi.1008354 (PMC7685438; doi:10.1371/journal.pcbi.1008354)
Supplement: Attachment — Submitted filename: Response_to_reviewers.docx [file pcbi.1008354.s020.docx]

William Noble, PhD

Deputy Editor

PLOS Computational Biology

Sushmita Roy, PhD

Associate Editor

PLOS Computational Biology

Dear Drs. Noble and Roy,

Thank you for handling our manuscript and organizing constructive reviews. Following the referee’s suggestions, we further revised the manuscript. New results are presented in the text, Fig 4, and the relating supporting information. We list detailed point-to-point responses to the review comments below. In addition to this, we corrected a few grammatical errors and clarified some sentences. We include a version where all changes are highlighted.

Reviewer #1: The authors have adequately addressed the substantive criticisms in my review, and I believe this will be an informative study for those planning transcriptomic studies in the mouse.

> We thank for the very positive and encouraging comment from the reviewer.

I have two minor comments:

- The title is now scientifically informative, but I continue to think the metaphor is only relevant to readers with similar cultural backgrounds. That this metaphor is repeated at conferences, likely involving the same limited in-group of investigators, is not helpful to a broad readership.

> We have now removed the metaphor from the title, but have retained it in the introduction. The reason is that it was a classical quote that has led to the establishment of the mouse phenotyping pipelines of today, as we have explained in the introduction. Hence, this quote has both, an important historical dimension, as well as a direct bearing to our study.

- In response to Rev 1, Item #5, and on Page 5/47 of the manuscript, the authors refer to "traditional phenotyping". I believe this needs further clarification, as referring to "tradition" does not address specific methods. Are serum biomarker assays, immunostaining, or ELISAs considered traditional methods, or do the authors primarily mean physiological traits?

> We have clarified the wording in the text, *i.e.*, we refer directly to the established phenotyping pipelines (ref 5) and their batteries of tests that include also blood analysis among many others.

Reviewer #2: I would like to thank the authors for taking into consideration the original comment and including a number of new analysis to address some of my concerns on applicability on understanding biological processes. The additional analysis include GO terms enrichment analysis and TF activity analysis. The GO-term enrichments are presented in terms of number of overlapping terms and they corroborate with the results from number of DEGs. As the authors point out- this is expected as GO enrichments are calculated based on DEGs.

> We appreciate the acceptance from the reviewer on the analyses in our previous revision.

What is still lacking from the paper is the relevance of the number of DEGs and GO terms in biological studies. While I agree that number is a good indicator of power and without identifying DEGs one is not able to perform downstream analysis but is getting more number of DEGs necessarily more helpful? What I meant in my original comment and still maintain is that usually researchers are interested in specific differences in biological processes or signalling pathways that are different between control and condition. Getting a large list of enriched terms or gene lists is not always useful for identifying function and the authors themselves have pointed this out in the ‘Tracking possible function” section. The authors shortlist their large list of GO enrichment by focusing on overlapping terms between the datasets and identifying 8 enriched terms. Could the authors tie this section clearly with their power analysis section? Would the eight enriched terms have not been identified, had the analysis have not been performed with a particular setup?

> We thank the referee for suggesting this additional analysis, since it turns out to confirm an important conclusion, namely the higher importance of increasing sample size rather than sequencing depth, which we had so far not specifically highlighted. But with this additional analysis, we have now decided to highlight this conclusion, in the abstract, the introduction and the discussion.
